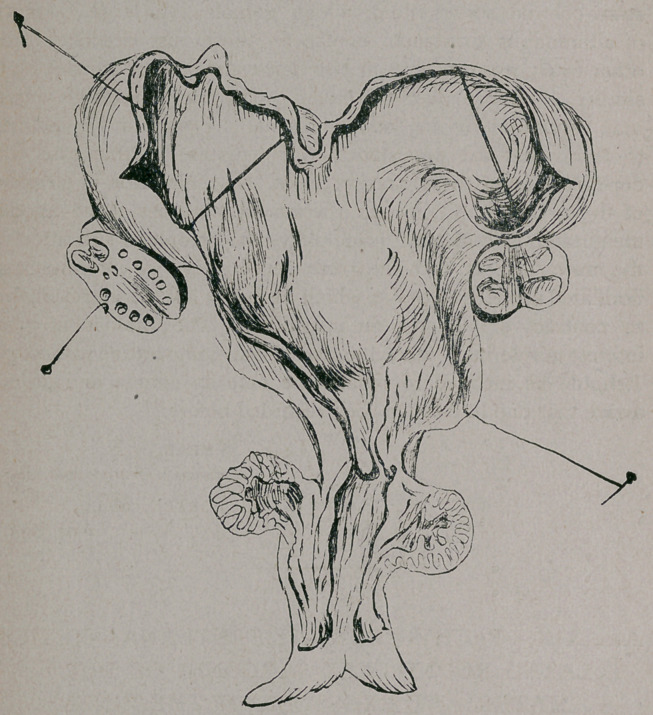# Chronic Endometritis in a Mare, with Enormous Distention of the Uterus

**Published:** 1881-10

**Authors:** L. E. Wheat, W. H. Porter

**Affiliations:** Scranton, Pennsylvania; New York


### ART. XVIII.—CHRONIC ENDOMETRITIS IN A MARE, WITH ENORMOUS DISTENTION OF THE UTERUS.

The history of this case dates back to February, 1875. Some time during that month the animal was obliged to wade through snow, while perspiring freely. That same evening she refused her food, and for several days following ate but little. The mare was not herself again until the following spring, when she was put to horse and turned out to grass.

Soon after this a discharge from the vagina was noticed, and being in heat she was again put to horse, which apparently arrested the discharge. Nothing further worthy of notice was observed for four years. In June, 1879, she was again put to horse, and twice during the month of August. After this she commenced discharging from the vagina a substance which was said to look like milk. The mare was treated for a while by the owner, as well as he knew how, but no improvement followed. In October, 1879, she was placed under the care of a would-be doctor, who diagnosticated leucorrhœa, and had the mare in charge until May, 1880, with no marked benefit.

In June, 1880, the mare was brought to me for examination and treatment, if any could be instituted with a prospect of improvement. Not able to make a thorough examination for the want of a proper speculum, I informed the owner to that effect, and was to call as soon as the proper instrument could be obtained. Messrs. Reynders & Co., of New York, enlarged one of Nott's three-bladed speculums, according to my directions, to meet the demand. July 28th, 1880, I made my first examination with the new instrument, and with it was enabled to see all that could be expected. At this time the mare was discharging from two to three quarts daily of thick yellowish purulent matter, having a very offensive odor.

By manipulation and a continued stream of warm water, I was able to dilate the os uteri. When the parts relaxed, the discharge became more profuse. After the discharge had somewhat abated, by the use of a lamp and reflector, I was enabled to see the whole of the vagina, and even into the uterine cavity. The floor of the vagina was considerably ulcerated. The os externum, and the portion of the uterine cavity brought into view, was also extensively ulcerated. I diagnosticated "chronic corporial endometritis," and gave an unfavorable prognosis.

Treatment, however, being desired by the owner, I ordered an alterative :

℞—Potass iodide, ʒ i.

Pulv. hydrastes canad., ʒ j.

Mix et ft. chart, No. 1 ; sig. ; use as directed.

To this was added a good, nutritious diet. The vagina to be injected twice daily with warm water, and the following to be injected after each hot water bath :

℞—Glycerine, ℥ iv.

Acid, tannic, ʒ i.

Morphin. sulphatis, gr. ij.

Mix ; sig., inject.

At other times I tried the following :

℞—Fld. ext. hydrastis, ℥ i.

Aqua grad., ℥ xvi.

Mix ; sig., inject.

To the ulcers that could be reached I applied, three times a week, a solution of nitrate of silver, gr. xxx., to aqua ℥ i.

Under this treatment the mare rapidly improved, and by October all discharge ceased. The mare was in good flesh, in fact quite fat. A tonic treatment, however, was continued. Medicated injections were discontinued, but the tepid vaginal baths continued. The mare continued quite well for some time, until the owner, thinking her recovery complete, without consulting me, drove her. Two weeks after driving, the vaginal discharge returned. The weather now being cold, the mare came down with a rheumatic attack. Vaginal and uterine treatment were not again instituted. From this time on the mare continued to lose flesh and strength until July, 1881, when I ordered her killed. At the necropsy nothing specially abnormal was noticed, except in the genito-urinary tract. The uterus contained about five gallons of very offensive pus, such as is occasionally found in the guttural pouches very inspissated and curdled slightly.

The genito-urinary organs were sent to the School of Histology in connection with the Columbia Veterinary College, where they were examined by Prof. Porter, and the following report rendered:

"The uterus was found greatly enlarged, the two cornua branching off from the body like two sacs. The ovaries were also enlarged. The os uteri was firmly closed, and it was with considerable difficulty that a probe could be passed either from the vagina into the uterus, or vice versa. The point of constriction appeared to be at the os internum. The uterus measured about thirty inches from os to fundus, and thirty-six inches from cornua to cornua. The uterus easily held two hundred ounces of water. The uterine walls were considerably thickened. The mucous membrane appeared to be completely destroyed, leaving the muscular tissue bare, or covered only by granulation tissue. Microscopically, the increased thickness of the uterine wall was found to be due, in great part, to an increase in the intermuscular fibrillated connective tissue, the muscular tissue showed very little, if any, increase in its elements; they appeared rather to be atrophied. This fact, I think, would explain the enormous dilatation which had taken place. The whole substance of the mucous membrane was found nearly destroyed, bearing little else than fibrous and muscular elements for the lining wall. This condition of the uterine substance differs somewhat from that found in the human subject where the uterus has been affected with "chronic corporeal endometritis," principally, however, in degree. It is rare, if ever, that the human uterus is extensively dilated, and probably there is never such a complete destruction of the uterine mucous membrane.

Both ovaries were the seat of extensive cystic degeneration. In one there was very little left of the normal ovarine tissue, it having been replaced by these large cysts, filled with a clear, straw-colored serous fluid, which contained a large quantity of albuminous substance, similar to those just described; the other ovary was the seat of two large and several smaller cysts similar to those just described. The vagina and os externum appeared to be nearly normal. I should be inclined to think that the persistence of the disease in this case was dependent upon these causes: First, the contraction or stricture of the os internum. Second, the almost total loss of the mucous membrane. Third, and principally, the atrophied condition of the muscular coat, with the great development of intermuscular fibrillated connective tissue, which probably left the uterus unable to contract or undergo an involution. The case is of great interest in veterinary medicine, and also in comparative pathology. I should be inclined to regard the case as unique, and am not aware that one like it has been recorded before."

L. E. WHEAT, V.S.,
*Scranton, Pennsylvania, and*
W. H. PORTER, M.D.,
*New York.*

## Figures and Tables

**Figure f1:**